# Meal replacement as a weight loss strategy for night shift workers with obesity: a protocol for a randomized controlled trial

**DOI:** 10.1186/s13063-022-06784-x

**Published:** 2022-10-08

**Authors:** Piumika Sooriyaarachchi, Ranil Jayawardena, Toby Pavey, Neil A. King

**Affiliations:** 1 Queensland University of Technology (QUT), Faculty of Health, School of Exercise and Nutrition Sciences, Brisbane, Queensland Australia; 2 Health and Wellness Unit, Faculty of Medicine, University of Colombo, Colombo, Sri Lanka; 3 Department of Physiology, Faculty of Medicine, University of Colombo, Colombo, Sri Lanka; 4 Nawaloka Hospital Research and Education Foundation, Nawaloka Hospitals PLC, Colombo, Sri Lanka

**Keywords:** Shift work, Obesity, Weight loss, Meal replacement, Low calorie

## Abstract

**Background:**

Shift work is considered a risk factor for a number of chronic health conditions including obesity. Weight reduction in obese patients lowers the risk for cardiovascular disease, diabetes, certain cancers, and mortality. Achieving a negative energy balance by providing low-calorie meal replacements is widely used for weight management. This study aims to evaluate the impact of a low-calorie “meal-replacement” on the weight and metabolic parameters of shift workers with obesity.

**Methods:**

This trial will be conducted in a parallel, randomized controlled design for a period of 8 weeks. A total of 44 shift workers with body mass index over 25 kg/m^2^ will be recruited after assessing eligibility. Participants will be randomly assigned to the test and control groups on a 1:1 ratio. The intervention group (*N* = 22) will be provided with a low-calorie (~200 kcal) meal replacement shake as dinner, and the control group (*N* = 22) will continue their habitual diets. The visits and the evaluations will be done as follows: screening (visit 0), 4 weeks (visit 1), and 8 weeks (visit 2). Anthropometric measurements will be taken at 0, 4, and 8 weeks. Body composition, biochemical parameters, dietary intake, and physical activity will be assessed during the first and the last visit.

**Outcomes:**

The primary outcome will be the proportion of participants that had a 5% body weight loss from baseline. The secondary outcomes will be post-intervention changes in other metabolic parameters.

**Discussion:**

To our knowledge, this is one of the first randomized controlled trials evaluating the effects of a meal replacement as the night meal for weight loss in shift workers with obesity. Moreover, improvement of metabolic parameters in shift workers will be an added benefit to this high-risk group.

**Trial registration:**

Australian New Zealand Clinical Trials Registry (ANZCTR) ACTRN12622000231741. Registered on 09 February 2022.

**Supplementary Information:**

The online version contains supplementary material available at 10.1186/s13063-022-06784-x.

## Introduction

The prevalence of shift work is increasing in modern society to cater to the complexity and flexibility of many operations that exceed the duration of a normal workday. Shift work is defined as work performed mostly outside of typical working hours [1]. It has been considered a major occupational hazard, and there is a plethora of research showing associations between shift work and chronic diseases [2]. Recent studies have shown that shift work was linked to an elevated risk of obesity, particularly abdominal obesity, with the risk being higher among long-term night shift workers [3].

Obesity is a major contributor to and a risk factor for increased morbidity and mortality due to a variety of diseases [4]. The global obesity burden has doubled since 1980 and the World Health Organization (WHO) predicts that one out of every five persons will be obese by 2025 [5]. Obesity has increased significantly in all South Asian countries [6]. The prevalence of overweight and obesity has increased by almost 40% from 1990 to 2013 in the Southeast Asian region [7]. The level of obesity in Sri Lanka has increased by several folds during the last two decades. According to the Sri Lankan body mass index cut-off (BMI ≥ 25 kg/m^2^), the prevalence of obesity in 2011 was 21.0% and 32.5% in men and women respectively [8]. In 2011, the obesity rate has been increased to 44.6% in males and 56.3% in females [9].

Unhealthy dietary habits, low physical activity, sleep deprivation, and disruption of the circadian rhythm have been proposed as the potential causes underlying weight gain among shift workers [10]. The sleep-wake cycle, thermogenesis, food intake, and lipids and glucose metabolism are reported to be influenced by circadian regulation [1]. Short sleep duration has been shown to lower leptin levels while increasing ghrelin levels, leading to an increase in appetite and weight gain [11]. In support of this view, several studies have shown that night shifts are associated with higher energy intake [12].

In addition, altered food habits also have a profound impact on peripheral circadian clock mechanisms [13]. Shift workers were found to consume more inflammatory diets than day workers, especially for dinner which creates a significant metabolic strain on the body (28). Shift work and snacking during evening work, in particular, are reported as common, with frequent sugar intake in the form of biscuits, soft drinks, and sandwiches that are easy to obtain [14]. Therefore, raising awareness about the effects of shift work on unhealthy food habits, and prescribing evidence-based interventions, may help shift workers’ health by preventing weight gain and associated metabolic consequences [15].

A loss of 5–10% of initial body weight is known to be effective in the treatment of many of the complications associated with obesity such as type 2 diabetes, hypertension, and hyperlipidemia [16]. Weight reduction is associated with increased performance and work capacity in obese individuals, thus contributing towards improving their quality of life [17]. Furthermore, following involvement in weight loss interventions, consistent significant increases in psychological indices such as self-esteem, depressive symptoms, body image, and health-related quality of life have been reported [18].

In comparison to standard energy-deficit diets that simply prescribe a reduction in energy intake, evidence shows that meal replacement diets result in greater weight loss [19], and better compliance [20] is more likely to provide an appropriate intake of essential nutrients, and have higher satisfaction and lower drop-out rates [21]. To the best of our knowledge, dietary intervention with a low-calorie meal replacement meal aimed to lose body weight and improve metabolic parameters has not been tested among night shift workers. Therefore, the present randomized controlled trial will be conducted to investigate the effectiveness of a low-calorie meal replacement dinner compared to a traditional dinner meal on metabolic parameters of shift workers with obesity.

## Methods

This protocol was written following the Standard Protocol Items: Recommendations for Interventional Trials (SPIRIT) checklist (Additional file 1) [22]. The schedule of trial enrollment, interventions, and assessments is presented in Table 1.

**Table 1:** Summarized study schedule at each visit in the clinical trial

	Study period			
	Enrolment	Allocation	Post-allocation	
**Time point**	−t_1_ (2 weeks)	0	Visit 1 (4 weeks)	Visit 2 (8 weeks)
**Enrolment**				
Eligibility screening	**×**			
Informed consent	**×**			
Allocation		**×**		
**Interventions**				
Meal replacement group			**×**	**×**
No meal replacement group			**×**	**×**
**Assessments**				
FBG		**×**		**×**
Serum HbA1c		**×**		**×**
Lipid profile		**×**		**×**
Blood pressure		**×**		**×**
Anthropometric measures		**×**	**×**	**×**
Body composition measures		**×**		**×**
FFQ/IPAQ		**×**		**×**

### Objectives and hypothesis

Hypothesis:

- Primary—prescribing a reduced caloric meal replacement at dinner will have beneficial effects on weight loss of night shift workers with obesity.
- Secondary—associated metabolic parameters (lipid profile, glycemic outcomes, and blood pressure) will be improved in the treatment group in comparison to the control group.

Objectives:

- Primary—to investigate if dinner meal replacement with a low-calorie meal replacement shake is an effective strategy to achieve a minimum 5% body weight loss in shift workers with obesity compared to conventional dinner
- Secondary—to evaluate the effects on anthropometric parameters, serum lipids, glycemic outcomes, and blood pressure

### Study design and setting

This study will be conducted as a parallel, randomized controlled clinical trial at the Nawaloka Hospital PLC, Colombo, Sri Lanka, for a period of 8 weeks, evaluating the effect of a meal replacement for dinner in obese shift workers.

### Sample size

A sample of 44 healthcare employees who engage in shift work will be recruited for the study after screening for eligibility against the inclusion and exclusion criteria mentioned below. The minimum sample size will be calculated based on the primary outcome, the proportion of participants achieving 5% weight loss after 8 weeks. Previous weight loss intervention trials predicted that 50% of individuals in the intervention group and 10% of participants in the control group would lose at least 5% of their baseline body weight after 8 weeks [23].. To detect this difference in the proportion of participants achieving 5% weight loss between the intervention and control groups, 17 participants were required in each group, to ensure 80% power at a 5% significance level. To accommodate a 20% dropout rate, we, therefore, needed to recruit 22 participants in each intervention group. Hence, a total of 44 adults with obesity (BMI >25 kg/m^2^) will be recruited for the study [24]. The formula used for sample size calculation is presented below:

$${n}_B=\left(\frac{P_{\textrm{A}}\left(1-{P}_{\textrm{A}}\right)}{K}+{P}_{\textrm{B}}\left(1-{P}_{\textrm{B}}\right)\right){\left(\frac{Z_{1-\alpha /2}+{Z}_{1-\beta }}{P_{\textrm{A}}-{P}_{\textrm{B}}}\right)}^2$$

*n*_B_ = Sample size in one arm

*P*_A_ = Proportion of subjects expected to achieve 5% weight loss at the end of 8 weeks in the placebo group (0.1)

*P*_B_ = Proportion of subjects expected to achieve 5% weight loss at the end of 8 weeks in the treatment group (0.5)

*Κ* = Sampling ratio (1:1)

*Z*_α_ = Critical value of the normal distribution at *α* (*α* is 0.05 and the critical value is 1.96)

*Z*_β_ = Critical value of the normal distribution at *β* (*β* is 0.2)

### Population

The study population will consist of obese healthcare employees in rotating shift work who provides health care services, either directly (e.g., doctors and nurses) or indirectly (e.g., helpers, laboratory technicians, medical waste handlers, security officers).

### Inclusion and exclusion criteria

#### Inclusion criteria

- Permanent employees aged 18–65 years
- Have been working shifts continuously for the past 12 months and continue during the whole study period (8 weeks).
- Working at least 3 night shifts/week
- Participants with BMI ≥25 kg/m^2^
- Not having any allergies to any of the known food ingredients, especially for milk and soya

#### Exclusion criteria

- Pregnant or lactating mothers
- Having known chronic disease conditions
- Having a known allergy history for milk or any of the ingredients in the meal replacement.
- History of any type of minor or major surgical procedure in the past 6 months.
- Currently on diet prescriptions or participating in regular physical activity sessions

#### Criteria for discontinuing or modifying the allocated intervention

- Subject’s request to discontinue the study
- Serious adverse events or unusual changes in clinical test results
- Principal investigator’s decision to terminate the study (low compliance rate, complications, or unable to continue the study due to various reasons)

There are no criteria for modifying allocated intervention.

### Assignment of intervention

#### Sequence generation

Participants satisfying the eligibility criteria will be allocated to control and intervention groups equally (1:1) using the simple random sampling technique. The randomization assignment will be generated by one independent investigator using a computer-generated random number sequence.

#### Concealment mechanism

Sequentially numbered, opaque, sealed, color-coded envelopes will be used to conceal the allocation sequences. To assign the patient to the appropriate group, the investigator who is involved in randomization must first open the subsequent color-coded envelope (treatment or control). The treatment and control groups shall be designated as either “A” or “B” and this allocation shall be kept in a sealed envelope in a secure place during the study.

#### Implementation

After all the participants have finished the screening and baseline assessments, participants will be informed of their randomized assignment. Eligibility evaluation and enrolment will be conducted by one independent investigator, while randomization will be done by another investigator.

#### Interventions

The test group will be provided with a 200-kcal meal replacement shake consisting of 20.0 g of protein, 4.5 g of fat, 18.2 g of carbohydrate, and 3.6 g of fiber with other vitamins and minerals. The ingredients and the nutritional composition of the product are presented in Additional file 2. The meal replacement will be donated by a pharmaceutical manufacturing company (Astron Limited). However, the manufacturer/sponsor organization will not be involved in any aspects related to the conduct of the study, including patient recruitment, follow-up, and analysis of data.

The meal replacement product will be in powdered form, and participants will be instructed to prepare the liquid shake by mixing 53 g of powdered meal replacement with 400 mL of water. They will be asked to consume one serving of the meal replacement shake to replace dinner for 5 days of the week for a period of 8 weeks. The intervention group will be given sufficient meal replacement tins with a label indicating the ingredients and the preparation method. Participants will be instructed not to consume any additional food between dinner in the evening, and breakfast the following morning. The breakfast and lunch meals will be recommended as normal meals (the meals that they habitually eat).

The control group will be asked to continue their usual dinner meals during the study period. In addition, both the groups will be given general dietary and lifestyle advice through the distribution of leaflets which includes basic dietary advice according to Sri Lankan food-based dietary guidelines. Both groups will be advised to continue their habitual physical activities.

### Study groups

The treatment group will receive the meal replacement shake for their dinner. The control group will be advised to continue their routine dinner.

### Study period

**Fig. 1:**
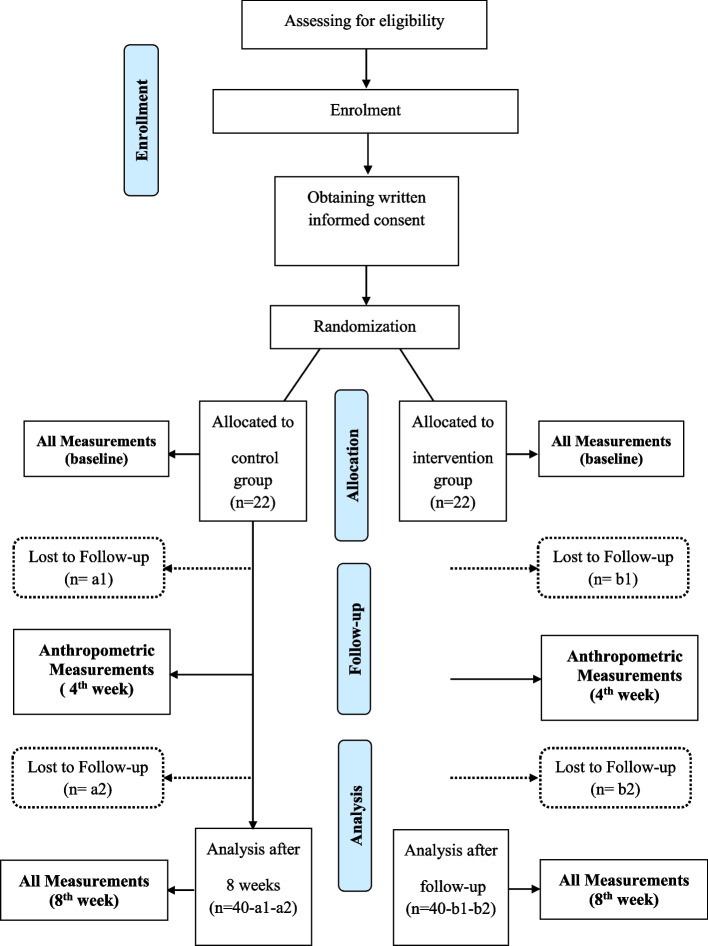
CONSORT flow diagram

The study lasts for 8 weeks, and periodic measurements will take place at screening (visit 0), 4 weeks (visit 1), and 8 weeks (visit 2) (see Fig. 1).

### Primary outcome

The primary outcome will be the proportion of participants that had a 5% body weight loss from baseline.

### Secondary outcomes

1. Changes in waist circumference, hip circumference, percentage of body fat, and fat-free mass
2. Change in systolic blood pressure and diastolic blood pressure
3. Change in lipid profile from baseline—total cholesterol, low-density lipoprotein (LDL), high-density lipoprotein (HDL), and triglycerides
4. Changes in glycemic control measures from baseline—fasting blood sugar, glycated hemoglobin (HbA1c)

### Procedures

#### Recruitment

Healthcare employees of the Nawaloka Hospitals PLC, Colombo, Sri Lanka, will be recruited voluntarily for this study by advertising through notices and distribution of leaflets with study information. Screening to identify eligible participants for the trial will be done by the research team and eligible participants will be given a detailed information sheet about the trial. The consent form will declare that participants are free to leave the study at any time, for any reason, without affecting their ability to receive future care and without being required to explain their decision. Further, the participant will have as much time as they need to think over and will have the chance to talk to the principal investigator or any other person before deciding whether or not to take part in the study. Next, the potential participants will be handed the consent form (Additional file 4) and given the chance to ask questions regarding the research. The informed written consent will be obtained by the principal investigator.

#### Study schedule

Anthropometric measures, blood pressure, fasting plasma glucose, serum HbA1c, and lipid profile will be measured at the time of recruitment of participants and will be repeated at the end of the 8th week. However, anthropometry measures will be again measured at the end of the 4th week additionally. The details of items that will be measured at every visit are described in Table 1.

### Measurement tools

#### Anthropometric measurements

Anthropometric measurements will be performed using calibrated equipment by trained personnel adhering to international recommendations. Height will be taken to the nearest 0.1 cm, as the maximum distance to the uppermost position on the head from heels, with the individual standing barefoot and in full inspiration using a calibrated stadiometer (Seca 213 portable stadiometer). Body weight will be measured to the nearest 0.1 kg using a calibrated digital weighing scale (Seca 813, Hamburg, Germany) with the participants wearing indoor light clothing and emptying the bladder. Waist circumference will be measured midway between the iliac crest and the lower rib margin at the end of normal expiration using a plastic flexible tape to the nearest 0 .1 cm. Similarly, the hip circumference will be measured at the widest level over the greater trochanters level to the nearest 0 .1 cm. BMI will be calculated as body mass in kilograms divided by height in meters squared (kg/m^2^) and waist-to-hip ratio by dividing the measured waist by hip circumference.

#### Body composition

Resistance (R) and reactance (X) will be assessed by bio-electrical impedance analysis (BIA) using a commercial portable device (Bodystat 1500, Bodystat Ltd, Isle of Man, British Isles) with hand-to-foot single frequency measurement while the subject remained supine. The participants will be asked to lie in the supine position, and then the electrodes of the BIA device will be placed on the right hand and foot. Metal objects will be removed from the body, and measurements will be taken at room temperature. To calculate fat-free mass and BF%, the resistance and reactance values will be applied to an ethnic-specific prediction equation developed for Asians [25].

#### Dietary measurements

A culturally validated Food Frequency Questionnaire (FFQ) will be used to obtain the habitual intake of calories, macronutrients, and micronutrients [26]. The energy content of each food component will be calculated using standard energy values for the portion sizes. The daily energy intakes are to be calculated using Nutri-Survey Software (EBISpro) modified with Sri Lankan food composition data.

#### Physical activity

Physical activity will be evaluated using the International Physical Activity Questionnaire (IPAQ) short version [27].

#### Biochemical analysis

A venous blood sample of 10–12 mL will be collected from each participant after overnight fasting. Blood glucose, total cholesterol, triglycerides, and HDL-cholesterol will be determined using a Cobas c501 auto analyzer using an electrochemiluminescent immunoassay (ECLIA, Roche Diagnostics). LDL cholesterol will be determined using the Friedewald formula. HbA1c will be evaluated by ion-exchange high-performance liquid chromatography.

#### Clinical examination

Seated blood pressure will be recorded on two occasions after at least a 10-min rest using a digital blood pressure monitor (Omron Healthcare, Singapore).

#### Compliance calculation

Powdered meal replacement will be dispensed to each participant at baseline, end of the 2nd, 4th, and 6th weeks. Participants will be instructed to return any unused meal replacement powder to the investigator at the end of the study. Compliance will be evaluated using the following formula:

$$\textrm{Compliance}\%=\frac{\left(\textrm{Distributed}\ \textrm{meal}\ \textrm{replacement}\ \textrm{quantity}-\textrm{Remaining}\ \textrm{meal}\ \textrm{replacement}\ \textrm{quantity}\right)}{\textrm{Distributed}\ \textrm{meal}\ \textrm{replacement}\ \textrm{quantity}}\times 100$$

In addition, researchers will phone participants periodically to monitor the consumption of the meal replacement. Each participant will be asked to record the meal replacement intake each day in a compliance assessment sheet provided at baseline. At each visit, these consumption records will be cross-checked with subjects. WhatsApp® groups will be created for communication with intervention and control groups.

### Relevant concomitant care permitted or prohibited during the trial

Participants will not be permitted to (1) engage in any other formal weight loss or exercise programs or clinical trials during the intervention, (2) use prescription or over-the-counter anti-obesity medications or supplements, and/or (3) undergo obesity treatment with bariatric surgery.

### Statistical analysis

Using SPSS version 23 (SPSS Inc., Chicago, IL, USA), parametric and non-parametric statistical tests will be applied for data analysis. Summary statistics of each group will be computed and presented as mean, standard deviation, and proportion. Distributions of continuous variables will be tested for normality using the Kolmogorov-Smirnov test. The non-parametric Mann-Whitney *U* test will be used for asymmetrical continuous variables. Using the paired *t*-test, the baseline and end of study characteristics, as well as the laboratory results of the groups, will be compared and a *P* value of <0.05 will be considered significant. The independent sample *t*-tests will be used (or suitable non-parametric test depending on data distribution) for the between-group differences and generalized linear mixed effects models for the analysis of longitudinal data.

Results will be presented as per-protocol and intention-to-treat analysis groups. The analysis will be carried out using the intention-to-treat (ITT) principle where patients’ data will be analyzed as members of the group they were randomized to, irrespective of their compliance with the treatment as specified in the protocol. A per-protocol (PP) analysis will be carried out including all patients who strictly adhere to the protocol in terms of following the treatment, availability of measurements, and absence of major protocol deviations. ITT analysis will use a generalized linear mixed model which can inherently deal with data missing at random. An effort will be made to reduce missing data to a minimum.

### Plans to give access to the complete protocol

The entire protocol will be available on the registry website and this paper provides the full protocol. Interested individuals should contact the study principal investigator (PS) if interested in other data or documentation of the study.

### Adverse effect evaluation

The meal replacement provides one-third of the Recommended Daily Allowance (RDA) of micronutrients in addition to 20 g of protein and a sufficient amount of fiber. Therefore, micronutrient deficiencies are very unlikely to occur. Furthermore, all participants will be thoroughly screened for any history of allergic reactions, particularly to milk products and other ingredients in the product. However, in the event of a probable adverse reaction, the following precautions would ensure timely identification and management of patients:

- Reporting—to monitor any potential adverse events, participants will have the opportunity to contact the researchers via WhatsApp®, 24 h daily.
- During follow-up visits, adverse events will be noted by history and examination and investigated in detail. All adverse effects observed will be documented in the case report form.
- A Data Safety Monitoring Board (DSMB) identified by the investigators will evaluate all adverse events at regular intervals. Refer to Additional file 3 for roles and responsibilities of DSMB.
- Management—in the event of an adverse reaction requiring in-hospital management, the facilities and expert management would be available at the Nawaloka Hospital PLC, Colombo, Sri Lanka.
- Termination of study—the complete clinical trial will be terminated prematurely if there is evidence that the safety of the trial participants can no longer be guaranteed, or if new scientific information arises during the clinical trial regarding the safety of the patients.

### Data collection

Data collection will be performed according to the standard operating procedures (SOPs) by the principal investigator and trained research assistants. The principal investigator will be responsible for establishing and maintaining contact with participants throughout the study. Contact information will be collected at enrollment, including alternate phone numbers and a contact person who will always be aware of the participant’s activities. The study coordinator will follow up with a call to reschedule if study appointments are missed.

### Data management

A participant identifier code for the data will be used so that data will not have the participant’s name associated with it. The key linking participant name and participant identifier code are kept in a secured location, with only the principal investigator having direct access to the list. Participant identifier codes will be used for all data entry and data analyses**.**

Data and documents will be locked and secured for 5 years, under the supervision of the principal investigator. Simultaneously, electronic copies will be stored in the Queensland University of Technology (QUT) Research Data Storage Service according to the QUT data management plan for the above prescribed period. Data will be entered by a minimum number of dedicated staff and saved in a dedicated computer with password protection. Samples will be stored in a secure facility, with measures taken to ensure that specimens are kept under correct and constant conditions at all times when in storage. Expert staff that is trained specifically in sample storage and transportation would ensure that all regulatory issues are properly handled.

### Frequency and plans for auditing trial conduct

To review the study’s conduct throughout the trial period, study team meetings, led by the principal investigator, will occur every week. A monthly progress report will be submitted to the DSMB.

### Dissemination of study findings

The results of the above study will be disseminated as publications and presentations in national and international journals or scientific meetings.

### Ethical considerations

The study protocol has been reviewed and approved by the Queensland University of Technology, Human Research Ethics Committee (UHREC approval no: 4878), and the institutional approval to conduct the study was taken from the Nawaloka Hospitals Research and Education Foundation, Colombo, Sri Lanka. The trial is also registered at the Australian New Zealand Clinical Trials Registry (ACTRN12622000231741). The study will be conducted in compliance with the Declaration of Helsinki and the good clinical practice guidelines. Any change in trial protocol will be notified to the relevant regulatory authorities and trial participants, with re-consent being taken from participants if required.

## Discussion

This manuscript describes the protocol for a clinical trial designed to evaluate the effects of having a low-calorie meal replacement dinner compared to a habitual dinner meal on the metabolic parameters of shift workers with obesity. To our knowledge, this is one of the first randomized controlled trials evaluating the effects of a low-calorie meal replacement dinner in shift workers with obesity.

The health sector is critical to any country’s social and economic development. Therefore, maintaining the well-being of healthcare employees is essential for improving the industry’s quality, productivity, and profitability. Healthcare employees should be physically and of sound mental health, in order to provide constant attention to patients’ requirements and ensure continuity of service in healthcare institutions. Most importantly, healthcare workers should be healthy role models for their patients. But, according to several studies, healthcare workers are no better than the general population when it comes to non-communicable diseases including obesity, diabetes, hypertension, and cardiovascular diseases [28].

Despite healthcare workers being well-informed about the causes and hazards of overweight and obesity, research in most countries has consistently indicated that they had a significantly greater risk of overweight, obesity, and metabolic syndrome than the general population. The prevalence of overweight and obesity among healthcare workers in Ghana ranged from 25.3 to 38.39% and 12.5 to 28.9% [29]. A study conducted in Botswana found that 34% of hospital employees had metabolic syndrome, 28.7% were obese, and 27.3% were overweight [30]. Another research on hospital employees found that late shift workers gained 4.3 kg more than day-shift workers (*P* = 0.02) after starting the current shift [31].

Habitual diet has a great influence on the well-being of healthcare workers. However, most of the employees in this industry find it extremely difficult to have a balanced meal while coping with their workload, and the demands of their work habits and associated lifestyle [32]. Several studies have shown an association between shift work and unhealthy eating habits, which could influence body composition and related health problems [33]. A study revealed that high energy density foods such as pastries, sweets, and sodas contributed to a significant portion of shift workers’ daily energy intake, with 18.1%, 20.7%, and 21.6% accounting for the morning, afternoon, and night shifts, respectively [34]. Also, the shift schedules often affect meal times and meal patterns. According to recent research, the timing of food intake has a substantial impact on metabolism and weight management [35].

When planning appropriate nutritional interventions for night shift employees, it’s also crucial to consider fatigue management and the unique requirements of night work [36], as food composition and meal distribution can have a significant impact on work performance and productivity [37]. In this study, we selected a low-calorie meal replacement approach for shift workers with obesity as a weight loss strategy to improve behavioral compliance, increase nutritional awareness, and its association with regular meals and less snacking [19]. Even in earlier research, participants who used meal replacements rated their dietary compliance and convenience higher than those who used traditional weight-loss regimens [38]. Therefore we believe that this would be a practical intervention to implement in the workplace.

Given the current interest in adopting low-calorie meal replacement meals to achieve weight loss in obese shift workers, no well-designed randomized control trials have been undertaken for a sufficient period to support or disprove this notion. Hence, the findings of this study will provide an alternative weight-loss for obese shift workers to lose their body weight without disrupting their work routine. Further, the findings of the current study would be useful in formulating new policies and designing new worksite weight loss interventions for employees’ obesity.

### Trial status

This article is in accordance with the study protocol version 2.0, dated 01 November 2021. Enrollment for the trial has not yet started.

## Supplementary Information

**Additional file 1.** SPIRIT checklist.

**Additional file 2.** Nutritional composition of the meal replacement product.

**Additional file 3.** Roles and responsibilities of DSMB.

**Additional file 4.** Consent form.

## Data Availability

Datasets generated and/or analyzed in this study will be available from the corresponding author on reasonable request after all identifiable information has been removed.

## Abbreviations

BIA: Bio-electrical impedance analysis
BMI: Body mass index
ECLIA: Electrochemiluminescent immunoassay
FFQ: Food frequency questionnaire
HDL: High-density lipoprotein
IPAQ: International Physical Activity Questionnaire
LDL: Low-density lipoprotein
QUT: Queensland University of Technology
RDA: Recommended Daily Allowance
SOPs: Standard operating procedures
SPIRIT: Standard Protocol Items Recommendations for Interventional Trials
WHO: World Health Organization
