# Supplementary material for: Meal replacement as a weight loss strategy for night shift workers with obesity: a protocol for a randomized controlled trial
Source: Trials. 2022 Oct 8;23:860. doi: 10.1186/s13063-022-06784-x (PMC9548175; doi:10.1186/s13063-022-06784-x)
Supplement: Supplementary file 2 — Additional file 2. Nutritional composition of the meal replacement product. [file 13063_2022_6784_MOESM2_ESM.docx]

**Supplementary table 1.** Nutritional composition of the meal replacement product

| **Nutrients** | **RDA** | **Per serving of 53g** | **% RDA per serving of 53g** |
| --- | --- | --- | --- |
| Energy (kcal) |  | 198 |  |
| Carbohydrates (g) |  | 16.5 |  |
| Protein (g) |  | 20.9 |  |
| Fat (MUFA 80%) (g) |  | 4.4 |  |
| Oligofructose (g) |  | 5.6 |  |
| **Vitamins** |  |  |  |
| Vitamin A (mcg) | 900 | 212 | 24 |
| Vitamin B1 (Thiamine HCl) (mg) | 1.2 | 0.13 | 11 |
| Vitamin B2 (Riboflavin) (mg) | 1.3 | 0.14 | 11 |
| Vitamin B3 (Niacinamide) (mg) | 16 | 1.75 | 11 |
| Vitamin B5 (Calcium D- Pantothenate) (mg) | 5 | 1.59 | 32 |
| Vitamin B6 (Pyridoxine HCl) (mg) | 1.7 | 0.54 | 32 |
| Vitamin B7 (D-Biotin) (mcg) | 30 | 3.18 | 11 |
| Vitamin B9 (Folic acid) (mcg) | 400 | 42.40 | 11 |
| Vitamin B12 (Cyanocobalamin) (mcg) | 2.4 | 0.27 | 11 |
| Vitamin C (Ascorbic Acid) (mg) | 90 | 10.60 | 12 |
| Vitamin D3 (mcg) | 20 | 10.60 | 53 |
| Vitamin E (mg) | 15 | 4.77 | 32 |
| Vitamin K (mcg) | 120 | 31.80 | 27 |
| **Minerals** |  |  |  |
| Calcium (mg) | 1300 | 318 | 24 |
| Magnesium (mg) | 420 | 53 | 13 |
| Zinc (mg) | 11 | 1.7 | 16 |
| Iodine (mcg) | 150 | 39.8 | 27 |
| Iron (mg) | 18 | 2.1 | 12 |
| Manganese (mg) | 2.3 | 0.2 | 9 |
| Sodium Chloride (mg) |  | 350 |  |

**Product ingredients**: Skimmed milk powder, Calcium caseinate, Sunflower oil powder, Oligofructose, Whey protein isolate, Sodium caseinate, Milk flavor, Maltodextrin, Cocoa powder; Isomaltulose, Minerals (Magnesium sulphate, Ferric pyrophosphate, Zinc sulphate monohydrate, Manganese sulphate monohydrate, Potassium iodide), Malt Extract, Sodium chloride, Caramel flavor, Sodium carboxymethyl cellulose, Xanthan. Gum, Ethyl vanillin, Caramel colorant, Vitamins (Vitamin E, Ascorbic acid, Vitamin D3, Niacinamide, Calcium D pantothenate, Vitamin A, Vitamin BL2, Vitamin K powder, Pyridoxine HCl, Ribo-5-phosphate sodium, Thiamin HCl, Folic acid, D Biotin, Sucralose
