# Supplementary material for: Meal replacement as a weight loss strategy for night shift workers with obesity: a protocol for a randomized controlled trial
Source: Trials. 2022 Oct 8;23:860. doi: 10.1186/s13063-022-06784-x (PMC9548175; doi:10.1186/s13063-022-06784-x)
Supplement: Supplementary file 3 — Additional file 3. Roles and responsibilities of DSMB. [file 13063_2022_6784_MOESM3_ESM.docx]

**TERMS OF REFERENCE**

**DATA & SAFETY MONITORING BOARD**

**Meal replacement as a weight loss strategy for night shift workers with obesity**

For the above study, the data and safety monitoring board (DSMB) will consist of the following members with different backgrounds covering endocrinology, nutrition, and medical research and statistics.

1. Dr. Sachith Abhayaratna, Specialist in Endocrinology & Senior Lecturer in Pharmacology, Faculty of Medicine, University of Colombo.
2. Dr Udara Abeywarne, Medical Officer, Medical Nutrition Unit, De Soyza Maternity Hospital for Women, Colombo, Sri Lanka.
3. Dr Amila Perera, Medical Officer, Medical Research Institute
4. Dr A. A Sunethra, Senior Lecturer, Department of Statistics, University of Colombo.

The main aim of the DSMB is to safeguard the interests of study participants, assess the safety and efficacy of the interventions during the study, and monitor the overall conduct of the study. The DSMB should make recommendations to the study working group through the principal investigators. The DSMB recommendations will then be shared with Ethics Review Committee (ERC) and other relevant regulatory bodies who will then make the decision whether to continue, suspend, modify, or stop a study.

The DSMB will be provided with any adverse event results so that they can see any emerging risks such as the frequency or severity of adverse events. Their monitoring should also include considering external factors to the study such as therapeutic or scientific developments that may have an impact on the safety of participants or the ethics of the study. The DSMB must protect the confidentiality of the study data and the results of monitoring.

**Their role and responsibilities are:**

- To understand the research protocol and the plans therein for data and safety monitoring
- To review any proposed modifications to the study prior to their implementation.
- To review interim summary reports including adverse event reports
- To inform the principal investigator on the acceptability of continued recruitment to the study and any concerns with regard to the safety or effectiveness of the drug

**OPERATIONS:**

The principal investigator will produce a report of the study monthly, including a progress report, which will be sent to each member of the committee (DSMB). They will evaluate the progress of the study, assessing safety data, data quality, timeliness, participant recruitment, accrual and retention, participant risk versus benefit, any other factors that can affect the study outcomes. If necessary, they will inform the PI to convene a meeting in addition to the scheduled meeting described below.

**MEETINGS:**

The committee will review reports via email. One main meeting will be convened by the PI at the end of 50% of the recruitment in addition to these email discussions.

**ACTIONS FOLLOWING MEETINGS**

Following meetings, the DSMB will send feedback through the principal investigators to the ERC. If there is any urgent potential safety or efficacy issues, then the DSMB should arrange a special meeting to discuss these issues. If the DSMB recommends that the study should be stopped or suspended, the PI will take the necessary actions to ensure that new recruitment to the study is stopped whilst the DSMB’s report is evaluated. The final decision to stop the study will be taken with the involvement of all relevant regulatory authorities, including the ERC.
