# Supplementary material for: Meal replacement as a weight loss strategy for night shift workers with obesity: a protocol for a randomized controlled trial
Source: Trials. 2022 Oct 8;23:860. doi: 10.1186/s13063-022-06784-x (PMC9548175; doi:10.1186/s13063-022-06784-x)
Supplement: Supplementary file 4 — Additional file 4. Consent form. [file 13063_2022_6784_MOESM4_ESM.pdf]

# Meal replacement as a weight loss strategy for night shift workers with obesity

## CONSENT FORM

**Part A - To be filled by the participant**

The participant should complete the whole of this sheet herself.

1. Have you read the information sheet? (Please keep a copy for yourself) YES/NO

2. Have you had an opportunity to discuss this study and ask any questions? YES/NO

3. Have you had satisfactory answers to all your questions? YES/NO

4. Have you received enough information about the study? YES/NO

5. Who explained the study to you? .......…………………………………………………

6. Do you understand that you are free to withdraw from the study at any time, without having to give a reason and without affecting your future medical care? YES/NO

7. Information held by the investigators relating to your participation in this study may be examined by other research assistants. All personal details will be treated as STRICTLY CONFIDENTIAL. Do you give your permission for these individuals to have access to your records? YES/NO

8. Have you had sufficient time to come to your decision? YES/NO

9. Do you agree to take part in this study? YES/NO

Participant's signature: …………………………. Date:……………………………

Name (BLOCK CAPITALS):
…………………………………………………………………………………………….

**Part B - To be filled by the investigator**

I have explained the study to the above volunteer and she/he has indicated her willingness to take part.

Signature of investigator: ……………………...... Date: …………………………....

Name (BLOCK CAPITALS):……………………………………………………………
